# Genetic Diversity and Population Structure of *Theileria annulata* in Oman

**DOI:** 10.1371/journal.pone.0139581

**Published:** 2015-10-15

**Authors:** Salama Al-Hamidhi, Mohammed. H. Tageldin, William Weir, Amira Al-Fahdi, Eugene H. Johnson, Patrick Bobade, Badar Alqamashoui, Albano Beja-Pereira, Joanne Thompson, Jane Kinnaird, Brian Shiels, Andy Tait, Hamza Babiker

**Affiliations:** 1 Department of Biochemistry, College of Medicine and Health Sciences, Sultan Qaboos University, P.O Box 35 Postal Code 123, Al-Khod, Sultanate of Oman; 2 Department of Animal and Veterinary Sciences, College of Agricultural and Marine Sciences, Sultan Qaboos University, P.O Box 34 Postal Code 123, Al-Khod, Sultanate of Oman; 3 Institute of Biodiversity, Animal Health and Comparative Medicine, College of Medical, Veterinary and Life Sciences, University of Glasgow, Glasgow, United Kingdom; 4 Ministry of Agriculture and Fisheries, Muscat, Oman; 5 Research Centre in Biodiversity and Genetic Resources (CIBIO), University of Porto, Rua Padre Armando Quintas 7, Vairão, 4485–661, Portugal; 6 Centre for Immunity, Infection & Evolution. Institutes of Evolution, Immunology and Infection Research, School of Biological Sciences, Ashworth Laboratories, University of Edinburgh, Edinburgh, United Kingdom; Onderstepoort Veterinary Institute, SOUTH AFRICA

## Abstract

**Background:**

Theileriosis, caused by a number of species within the genus *Theileria*, is a common disease of livestock in Oman. It is a major constraint to the development of the livestock industry due to a high rate of morbidity and mortality in both cattle and sheep. Since little is currently known about the genetic diversity of the parasites causing theileriosis in Oman, the present study was designed to address this issue with specific regard to *T*. *annulata* in cattle.

**Methods:**

Blood samples were collected from cattle from four geographically distinct regions in Oman for genetic analysis of the *Theileria annulata* population. Ten genetic markers (micro- and mini-satellites) representing all four chromosomes of *T*. *annulata* were applied to these samples using a combination of PCR amplification and fragment analysis. The resultant genetic data was analysed to provide a first insight into the structure of the *T*. *annulata* population in Oman.

**Results:**

We applied ten micro- and mini-satellite markers to a total of 310 samples obtained from different regions (174 [56%] from Dhofar, 68 [22%] from Dhira, 44 [14.5%] from Batinah and 24 [8%] from Sharqia). A high degree of allelic diversity was observed among the four parasite populations. Expected heterozygosity for each site ranged from 0.816 to 0.854. A high multiplicity of infection was observed in individual hosts, with an average of 3.3 to 3.4 alleles per locus, in samples derived from Batinah, Dhofar and Sharqia regions. In samples from Dhira region, an average of 2.9 alleles per locus was observed. Mild but statistically significant linkage disequilibrium between pairs of markers was observed in populations from three of the four regions. In contrast, when the analysis was performed at farm level, no significant linkage disequilibrium was observed. Finally, no significant genetic differentiation was seen between the four populations, with most pair-wise F_ST_ values being less than 0.03. Slightly higher F_ST_ values (G_ST_’ = 0.075, θ = 0.07) were detected when the data for *T*. *annulata* parasites in Oman was compared with that previously generated for Turkey and Tunisia.

**Conclusion:**

Genetic analyses of *T*. *annulata* samples representing four geographical regions in Oman revealed a high level of genetic diversity in the parasite population. There was little evidence of genetic differentiation between parasites from different regions, and a high level of genetic diversity was maintained within each sub-population. These findings are consistent with a high parasite transmission rate and frequent movement of animals between different regions in Oman.

## Introduction

Theileriosis is a tick-borne disease of domestic and wild animals and is economically devastating to livestock production in tropical and subtropical regions of the Old World [1–3]. In Oman, theileriosis is caused by infection with the protozoan parasite *Theileria annulata*, which is transmitted by ticks of the genus *Hyalomma* and infects cattle. In addition, *T*. *lestoquardi* and *T*. *ovis* are the common *Theileria* species associated with theileriosis in small ruminants within Oman [3, 4]. The *Theileria* parasite undergoes haploid asexual replication in the vertebrate host followed by a diploid sexual phase in the tick vector [5]. The parasites enter the host during tick feeding and rapidly invade host leukocytes, primarily of the myeloid lineage [6]. Ultimately, merozoites are produced and released from the infected leukocyte as it is destroyed. Free merozoites invade erythrocytes and develop into piroplasms [6]. Animals which survive acute disease become carriers of *Theileria* piroplasms and play an important role as reservoirs for the maintenance of the parasite population [7]. Identification of carrier animals is, therefore, of utmost importance in epidemiological studies for inferring infection risk and for implementation and monitoring of control programs [8].

The population structures of many protozoan parasites have been suggested to be predominantly clonal [9, 10]. However, surveys of a number of vector-borne parasite species such as *T*. *annulata* have demonstrated a high level of genetic diversity and evidence of panmixia [4]. Genetic diversity underpins the phenomena of ‘antigenic variation’ and diversity that allow parasites evade the host immune response [11, 12]. A major mechanism underlying generation of genetic diversity is chromosomal recombination events during sexual reproduction and this has been documented in several apicomplexan species, such as *Plasmodium falciparum* [13]. Other mechanisms such as isolation, genetic drift and mutation play a role in maintaining genetic diversity [12]. Acquisition of genetic diversity is beneficial to the long-term survival of parasite species, and often complicates the establishment (or compromises the effectiveness) of control measures. Thus, it is important to appreciate the extent of genetic diversity in parasite populations in order to understand how they may respond to control measures such as vaccination and drug treatment [12].

A number of studies have examined the genetic diversity and population structure of *Theileria* parasites between and within countries [4]. The question of whether the parasites in each country form distinct non-overlapping populations or if there is gene flow between parasite populations has practical implications for control strategies. For example, in the case of development of drug resistance, a resistant genotype arising in one population as a result of local drug usage would not spread to other populations if the parasites were reproductively isolated and there was no emigration of genotypes [14]. Similarly, an effective multivalent vaccine capable of providing cross-protection against multiple genotypes that circulated in one population may not be successful in another genotypically distinct population.

Attempts have been made to examine the population genetic structure of *T*. *annulata* and *T*. *parva* using polymorphic micro- and mini- satellites [15, 16]. A high level of diversity has been observed among *T*. *annulata* both between and within countries, and evidence of sub-structuring has been detected and positively correlated with geographical distance. Similarly, a high level of diversity was seen among *T*. *parva* genotypes in the Lira and the Kayunga (in Uganda) populations, with evidence for geographical sub-structuring between populations in each area [15].

In the present study, ten polymorphic micro- and mini-satellites distributed across the four chromosomes of the *T*. *annulata* genome have been used to examine diversity and population structure of this parasite in Oman. The number and frequency of alleles at each locus, the level of linkage disequilibrium (LD) between pairs of loci and the genetic distance within and between *T*. *annulata* populations in four regional governorates of Oman were examined. This data was compared to similar datasets previously obtained in Tunisia and Turkey. The results provide the first data on the population structure of *T*. *annulata* in the Arabian Peninsula.

## Methods

### Parasite samples and DNA preparation

**Fig 1:**
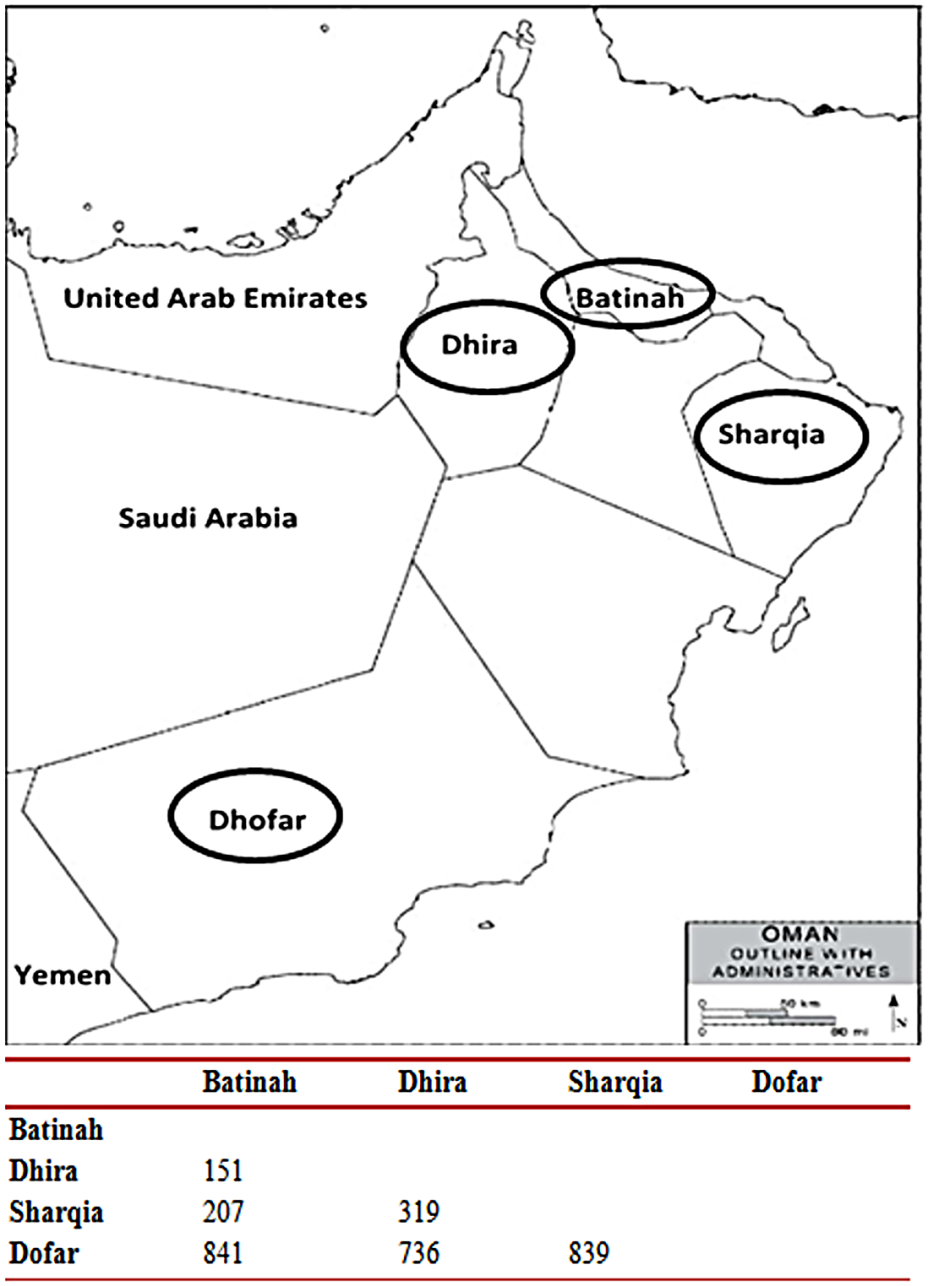
Locations of collection sites in four regions in Oman; below table represent geographical distance matrix in km between regions. The study field surveys and samples collection were carried out in accordance with the regulations of the Sultan Qaboos University Committee for Animal Ethics. The field surveys did not involve endangered or protected animal species: blood samples were collected by a veterinarian while animals were manually restrained; no tranquillisers or short-acting anaesthetics were used. Appropriate equipment was used for blood sample collection. No institutional approval was needed, as per Sultan Qaboos University ethics committee such an approval is only required for small animals. The sampling procedures and number of animals to be sampled were approved by the Ministry of Agriculture and Fishery, Oman, as part of obtaining the field permit.

454 blood samples were collected from cattle not showing clinical signs in four regional governorates in Oman: Batinah (n = 81), Dhira (n = 121), Sharqia (n = 25), and Dhofar (n = 227). Samples were collected from approximately 20 randomly selected farms in each governorate with around fifty animals per farm sampled. The sample size represents 10% of the herd on each farm; in a few instances when the number of animals collected on a farm was below this size, additional samples were collected from other randomly selected herds. The location and distance between each of the four governorates is shown in Fig 1.

The climate in Batinah, Dhira and Shariqa is dry throughout the year. However, the climate of the southern parts of Oman (Dhofar) is markedly different from that of the rest of the country due to the effects of the sustained summer monsoon rains that fall between June and September. During the monsoon, the weather is wet and slightly warm (average 31°C) to cold (average 23°C) and because of this climate, the Dhofar region has the only natural pasture-land in Oman. During the monsoon, the animals graze on natural pastures during the day and are herded into shelters in the evening. However, cattle in other parts of Oman are kept exclusively on farms and movement from farm to farm is not an uncommon practice.

Nine species of ixodid ticks are found on cattle in different regions in Oman. These are *Amblyomma variegatum*, *Rhipicephalus (Boophilus) annulatus*, *Hyalomma anatolicum*, *H*. *dromedarii*, *H*. *detritum detritum*, *H*. *excavatum*. *H*. *impeltatum*, *H*. *rufipes* and *R*. *camicasi*. [17].

Approximately 10 ml of peripheral blood was collected from each animal using tubes containing EDTA. DNA was extracted using the Qiagen QIAamp DNA mini Kit (Qiagen, Germany) following the manufacturer’s instructions and stored at -20°C.

### Identification of *T*. *annulata*

Pan-*Theileria* primers were designed, targeting the 18S rRNA locus: F 5'-GGCGTTTATTAGACCTAAAACCAAAC-3' and R 5'-TTTGAGCACTCTAATTTTCTCAAAGT-3'. 2 μl of template DNA was amplified in a 25 μl reaction mixture. PCR conditions were as follows: denaturation at 95°C for 5 minutes, 35 cycles at 95°C for 30 seconds, 58°C for 30 seconds, and 65°C for 30 seconds, followed by a final extension step of 5 minutes at 65°C. The amplified products were then analysed on a 2% ethidium bromide pre-stained agarose gel. To differentiate between the common *Theileria* species in Oman (*Theileria annulata*, *T*. *lestoquardi* and *T*. *ovis*), PCR products were subjected to restriction fragment length polymorphism (RFLP) analysis with *HpaII* restriction enzyme (Biolab, UK). Electrophoresis was used to separate the fragments that resulted from the endonuclease incubation. Fragment sizes representative for the different *Theileria* species are as follows: *T*. *annulata* (GenBank: KF559356.1) 357, 94, 39 bp; *T*. *lestoquardi* (GenBank: KJ458988) 278, 94, 79, 39 bp; and *T*. *ovis* (GenBank: KP019206.1) 326, 136, 39, 35 bp.

### Analysis of micro- and mini-satellite loci

A panel of ten polymorphic micro- and mini-satellite markers designed for the genetic analysis of *T*. *annulata* [16] was used to genotype each sample. The forward primer in each marker set was labeled with a fluorescent dye at the 5' end. The 25 μl PCR mixture contained 2 μl of template DNA and 1μl of each forward and reverse primer (10 pmol) and the DNA region amplified using Biolab Taq DNA polymerase (Biolab, UK) under the following conditions: denaturation at 95°C for 5 minutes, 32 cycles at 95°C for 30 seconds, 42–62°C for 30 seconds and 65°C for 30 seconds, followed by a final extension step of 5 minutes at 65°C. The amplified products were observed on 2% ethidium bromide pre-stained agarose gel to determine the efficacy of PCR amplification. PCR products were then denatured and subjected to capillary electrophoresis on an ABI3130 xl Genetic Analyser (Applied Biosystems, UK). DNA fragment sizes were analysed relative to ROX-labeled GS500 ROX size-standard (Applied Biosystems) using Genemapper software (Applied Biosystems). The use of this technology facilitated the discrimination of multiple amplicons in a single reaction with a resolution of 1 base pair (bp). Multiple products from a single PCR reaction indicated the presence of a mixture of genotypes. To determine the relative concentration of each allele/amplicon, the area under each peak was assessed. In this way, the predominant allele at each locus was identified for each sample and this data was combined to generate a multi-locus genotype (MLG) that represents an estimate of the most abundant genotype in each sample. As peak area is essentially a continuous variable, it was always possible to identify the most abundant allele in any sample.

### Data analysis

Tandem software [18] was utilised to facilitate consistent allele-calling, and the Excel microsatellite toolkit used for similarity comparison of the MLGs [19]. Genetic diversity parameters were calculated for the entire dataset using GenAlex v6.5 [20, 21]. This included determining the number of alleles per locus (A), and expected heterozygosity (H_e_). These two parameters were used to assess the level of polymorphism at each locus and determine diversity overall and within the sub-populations. H_e_ was calculated using the formula for ‘unbiased heterozygosity’ also termed haploid genetic diversity, H_e_ = [n/(n-1)][1-∑p^2^] where *n* is the number of isolates and *p* the frequency of each different allele at a locus [22]. To determine whether the *T*. *annulata* populations in different regions comprised a single panmictic population with a high degree of genetic exchange, Multilocus linkage disequilibrium (LD = non-random association of allele among loci) of the alleles at pairs of loci was measured using the standard index of association (I^S^_A_), and pooling all samples from four regions or samples from each region. Both I^S^_A_ and variance of data were calculated using the program LIAN version 3.5 [23]. The software tests for independent assortment of alleles by determining the number of loci at which each pair of MLGs differs, and from the distribution of mismatch values a variance V_D_ (the variance of the number of alleles shared between all pairs of haplotypes observed in the population) is calculated, which is then compared with the variance expected for LE, termed V_e_. The null hypothesis that V_D_ = V_e_ is tested by a Monte Carlo simulation and a parametric method and the results provide 95% confidence limits, which are denoted L_MC_ and L_PARA_, respectively. If there is limited or no association between alleles at different loci, indicating panmixia, a value close to zero is obtained, whereas if association is detected, a value significantly greater than 0 is obtained, indicating non-panmixia [23]. The variance of pair-wise difference (V_D_) between the data and that predicated for panmixia (V_e_) and L (L_MC_ & L_PARA_), were calculated in order to test the hypothesis of panmixia. Therefore when the V_D_ value exceeds the L value, LD is indicted and the null hypothesis of panmixia is discarded. When the V_D_ is less than L, LE is indicated and the null hypothesis of panmixia is accepted.

Population differentiation was examined by estimation of F statistcs, using the Fstat computer package Version 2.9.3.2. Two estimators of F_ST_ (G’_ST_ and θ) [24–26] were used to estimate genetic differentiation between sub-populations. The structure of the *T*. *annulata* population in Oman was investigated by analysis of molecular variance (AMOVA) [27]. Principal Co-ordinate analysis (PCoA) was used to visualise the relationship between MLGs using GeneAlex6.

### Multiplicity of infection

Multiplicity of infection was defined as “presence of multiple genotypes per infection” detected by the presence of more than one allele at a locus. To avoid over-estimation of rare alleles, only minor alleles having peak height >33% of the corresponding predominant allele are accepted. This eliminates spurious peaks in the vicinity of major peaks, which if unaccounted for can overestimate the presence of rare alleles thus artificially inflating the calculated multiplicity of infection [4]. The mean number of alleles across the ten loci in each sample was calculated and this index value represented the multiplicity of infection within each sample. The overall mean for the index value for each sample was then calculated to provide the average multiplicity of infection for each region.

To determine whether the host variables, i.e. age, sex and breed could explain the multiplicity of infection in individual hosts. The analysis of co-variance (ANCOVA) is an alternative term for linear regression modelling using a single continuous explanatory variable along with one or more conditional factors. This was achieved by comparing the average number of alleles present at each locus in an individual sample against the three host parameters using the statistical software package XLSTAT 2006

## Results

### Prevelance of *T*. *annulata* in different regions in Oman

The screening of cattle blood samples (n = 447) using 18S rRNA gene primers, revealed a total of 323 (72.3%) *T*. *annulata* positive cases for all four regional governorates in Oman. No other *Theileria* speceis was detected. The proportion of *T*. *annulata* infected individuals among the examined cattle varied significantly between the four governorates (P = 0.0000803) and was highest in Dhofar (n = 171 [80.3%]) followed by Batinah (n = 58 [74.4%]), Sharqia (n = 26 [72.2%]) and Dhira (n = 68 [56.7%]).

### Marker diversity and allelic variation

**Fig 2:**
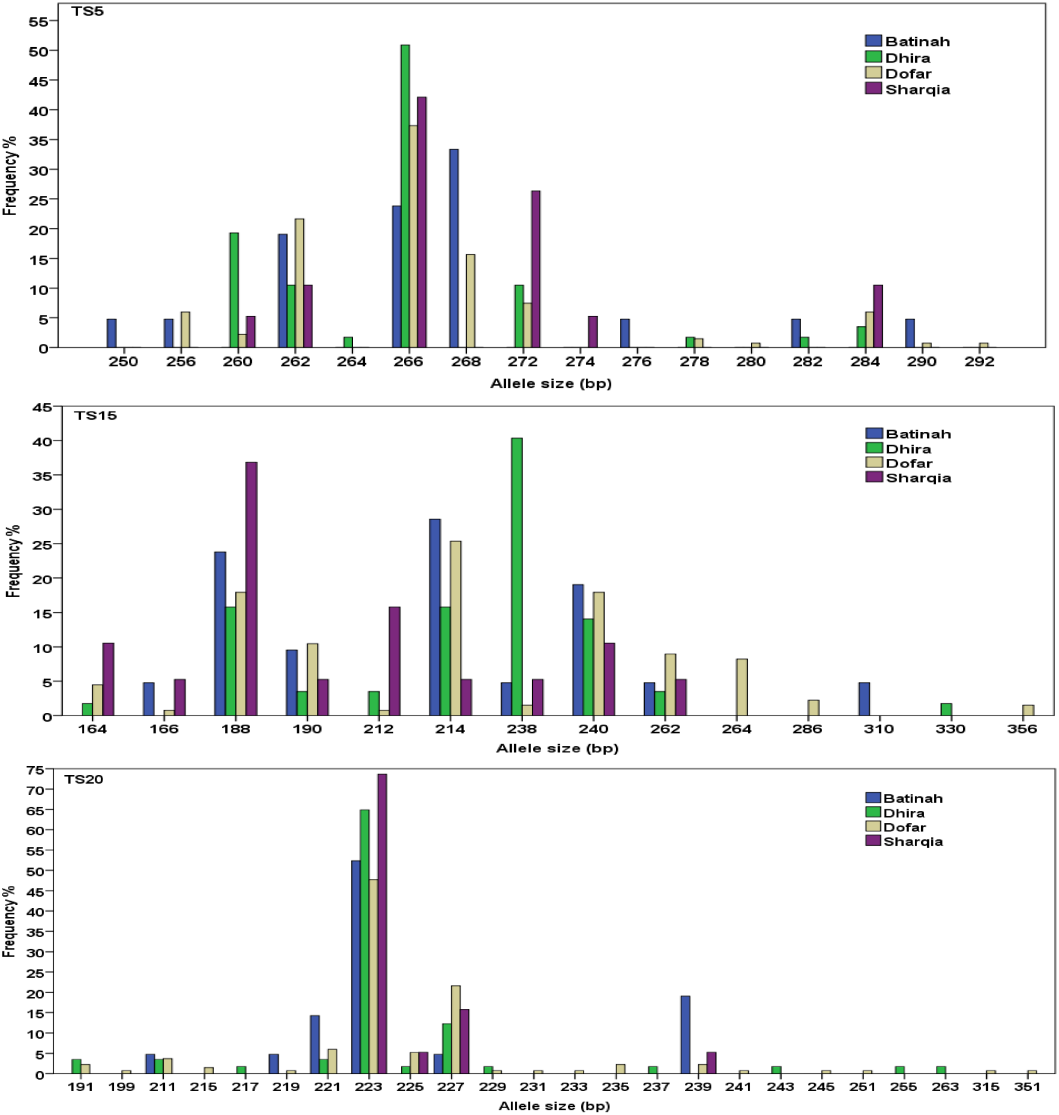
The frequency of alleles for three representative markers (TS5, TS15 and TS20) in four regional parasite populations in Oman. The size of each allele (in bp) is given on the x- axis.

Out of 323 positive samples, 310 *T*. *annulata* isolates from the four governorates were succesfully typed using ten micro- and mini-satellite markers. Each marker was found to be highly polymorphic in samples from all four regions, with the overall number of alleles per locus ranging from 16, for TS15 to 51 for TS6 (Table 1). Although there were a number of private alleles specific to each parasite population, broadly similar allele frequencies were observed in each region (for example, TS5 and TS15, Fig 2). However, a higher number of low frequency private alleles were seen in Dhofar, such as with marker TS20 (Fig 2). This can be attributed to a larger positive sample size or higher population density/transmission frequency in this area, which was more than twice that found in the other regions.

**Table 1:** Allelic diversity and unbiased heterozygosity (*He*) at 10 micro- and mini-satellite loci among 310 *T*. *annulata* isolates in Oman.

	n	TS5	TS6	TS8	TS9	TS12	TS15	TS16	TS20	TS25	TS31	Average *He*
**Minimum number of alleles**	310	1	1	1	1	1	1	1	1	1	1	
**Maximum number of alleles**	310	8	8	10	8	11	6	9	9	8	9	
**No PCR amplification**	310	11	30	10	5	19	4	35	14	29	39	
**Total alleles number**	310	19	51	48	39	49	16	20	27	18	35	
**Diversity in Batinah**	45	0.824	0.976	0.976	0.9	0.819	0.848	0.776	0.695	0.79	0.933	0.8537
**Diversity in Dhira**	67	0.692	0.934	0.971	0.928	0.91	0.777	0.648	0.568	0.835	0.935	0.8198
**Diversity in Dhofar**	173	0.782	0.789	0.873	0.901	0.932	0.849	0.724	0.72	0.725	0.87	0.8165
**Diversity in Sharqia**	25	0.766	0.912	0.965	0.953	0.86	0.848	0.895	0.45	0.83	0.848	0.8327
**Overall diversity**	310	0.782	0.893	0.942	0.932	0.949	0.871	0.747	0.655	0.764	0.919	0.8307

### Population diversity and linkage disequilibrium

More than one allele per locus was common in all field samples, therefore multi-locus genotypes (MLGs) were constructed, based on the predominant allele present at each locus, and used to generate the dataset for population genetic analysis. The MLG dataset (Table 1, S1 Table) was used to measure population genetic indices such as heterozygosity, linkage disequilibrium and population differentiation. Since *Theileria* is haploid within the bovine host and heterozygosity cannot be observed directly, the estimated heterozygosity (*H*_*e*_) was calculated using the predominant allele dataset for each marker and averaged across all ten loci. The average heterozygosity within each region was generally high, ranging from 0.816 within Dhofar to 0.854 in Batinah.

To determine whether the *T*. *annulata* populations in the different regions comprised a single panmictic population with a high degree of genetic exchange, the level of linkage equilibrium of alleles at pairs of loci was measured using the standard index of association (I^S^_A_). When the samples from the four regions were treated as a single population (i.e. Oman), an I^S^_A_ value of 0.019 and a V_D_ value (1.4595) greater than L (1.29) was obtained, indicating LD (Table 2).

**Table 2:** Linkage equilibrium among *T*. *annulata* populations in Oman and comparison of parasites in Oman, Tunisia and Turkey.

	I_A_^S^	V_D_	L_MC_	L_PARA_	Linkage
**Oman**	0.019	1.4595	1.29	1.29	LD
**Batinah**	-0.0131	1.0344	1.4076	1.3806	LE
**Dhira**	0.0219	1.5537	1.4509	1.4378	LD
**Dhofar**	0.0231	1.7435	1.5167	1.5129	LD
**Sharqia**	0.0337	1.5627	1.4451	1.4342	LD
**Oman, Tunisia & Turkey**	0.0238	1.1099	0.9317	0.9312	LD
**Oman & Tunisia**	0.0224	1.1846	1.0075	1.0092	LD
**Oman & Turkey**	0.0238	1.1099	0.9309	0.9312	LD

To test the hypothesis of geographical sub-structuring, the I^S^_A_, V_D_ and L values were calculated separately for samples in each region. The I^S^_A_ value for Dhira, Sharqia and Dhofar was 0.0219, 0.0337 and 0.023, respectively. The V_D_ values were greater than the L value, indicating LD in all regions (Dhira, Sharqia and Dhofar) except Batinah.

### Population sub-structuring

#### F_ST_ analysis

To measure the level of genetic differentiation between populations, the F_ST_ value was estimated. Two estimates of F_ST_ (G’_ST_ and θ) [24, 26] were calculated and gave consistent results for all combinations of the populations analysed (Table 3). Based on these calculations, a low amount of differentiation (G’_ST_ = 0.033 and θ = 0.04) was evident among the four sites in Oman when considered together and similarly, a low level of differentiation was indicated when the populations from each region were compared with each other in a pair-wise manner (Table 3).

**Table 3:** Pair-wise F_ST_ estimates among *T*. *annulata* populations in Oman, as well as between Oman, Tunisia and Turkey.

		F_ST_	
	N	θ	θ SE
**Oman**			
**Batinah, Dhira, Dhofar & Sharqia**	231	0.04	0.007
**Batinah & Dhira**	78	0.026	0.013
**Batinah & Dhofar**	155	0.038	0.015
**Batinah & Sharqia**	40	0.02	0.012
**Dhira & Dhofar**	191	0.045	0.009
**Dhofar & Sharqia**	153	0.043	0.01
**Dhira & Sharqia**	76	0.027	0.015
**Oman, Tunisia & Turkey**	488	0.07	0.018
**Oman & Tunisia**	429	0.063	0.019
**Oman & Turkey**	290	0.103	0.024

Furthermore, the parasite population in Oman was compared to parasite populations in Turkey and Tunisia using the same ten loci [4]. The F_ST_ estimated for the three populations (Turkey, Tunisia and Oman), demonstrated moderate differentiation between parasite populations between Oman and Tunisia (G’_ST_ = 0.063 and θ = 0.063) and a slightly higher level of differentiation between Oman and Turkey (G’_ST_ = 0.102 and θ = 0.103) (Table 3).

### PCoA and AMOVA analysis

**Fig 3:**
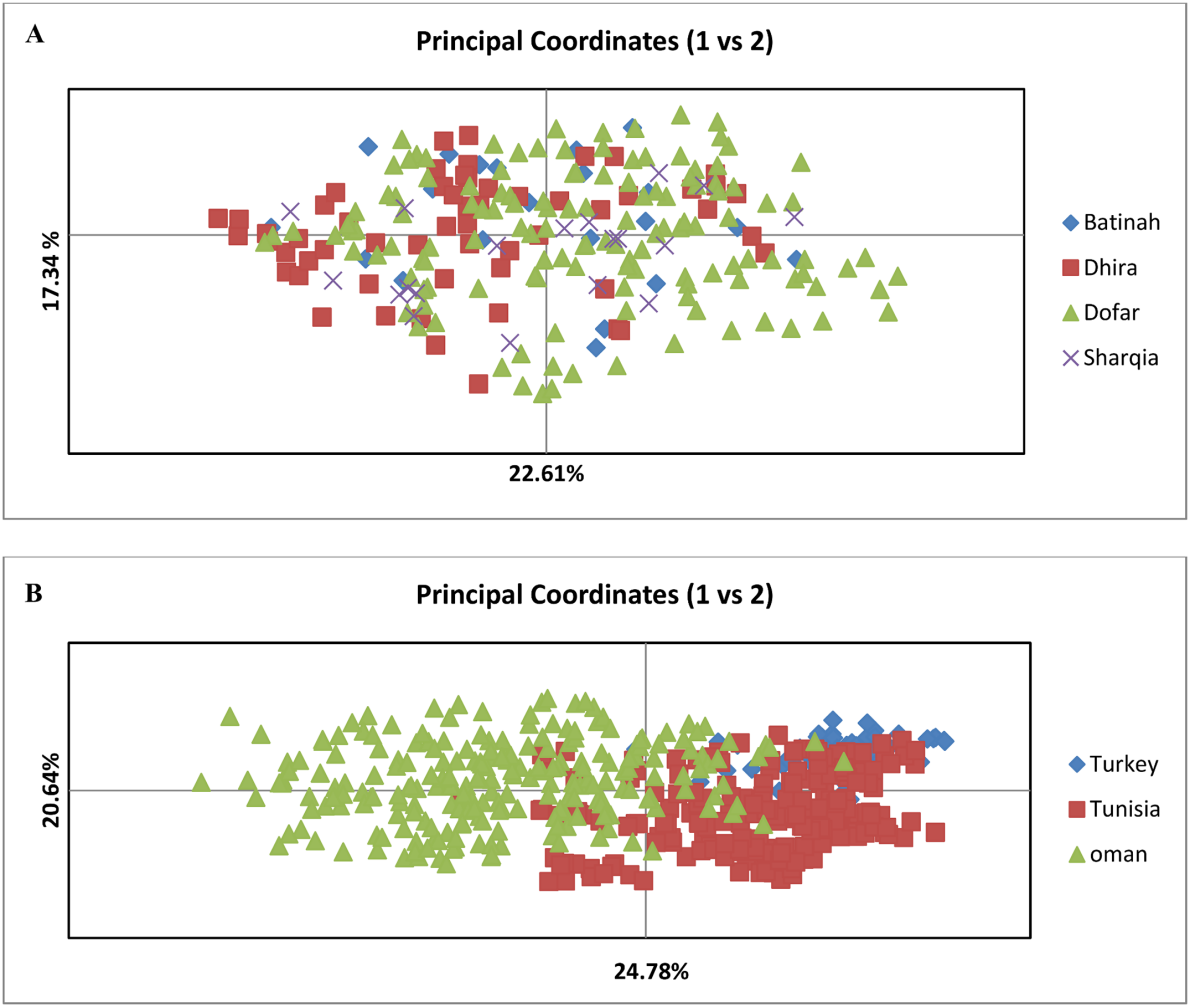
Principal Co-ordinate analysis (PCoA) of *T*. *annulata* populations from Oman, Turkey and Tunisia. PCoA was performed on the multi-locus genotype data representing each of the populations sampled. The proportion of the variation in the dataset explained by each axis is indicated in parenthesis.

The low level of differentiation between parasite populations across four regions in Oman was supported by PCoA analysis (Fig 3) as no clear geographical clustering was evident. The amount of molecular variation obtained represented by the first and second axes was 22.61% and 17.34%, respectively. The finding of genetic differentiation between populations from the three countries (Oman, Turkey and Tunisia) (see above) was supported by PCoA analysis (Fig 3), where data from samples with a complete MLG profile was analysed together with Turkish and Tunisian samples. The clustering on the PCoA indicates that Turkish and Tunisian populations are distinct from the Omani population, with the Tunisian population being closer to the Omani than the Turkish parasite population, indicating geographic sub-structuring.

Analysis of molecular variance (AMOVA) was used to assess the population structure of *T*. *annulata* in three countries by partitioning variation among and within parasite sub-populations. Most of the genetic variation (93%) was contained within sub-populations with only 7% explained by differences between sub-populations. This suggests the occurrence of a high rate of cross-mating and recombination within the parasite population as a whole.

### Multiplicity of infection (MOI)

**Fig 4:**
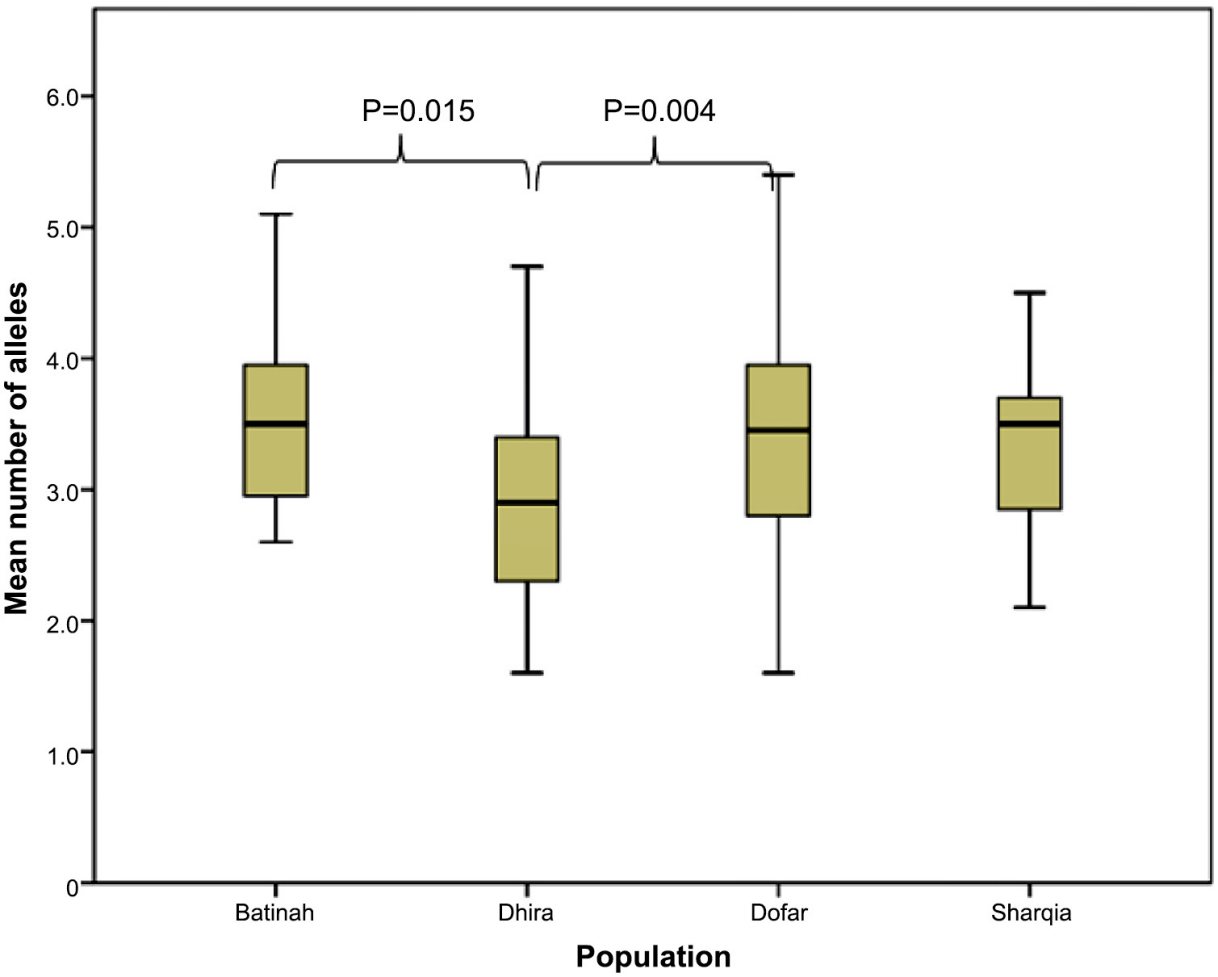
Box plot of mean number of alleles in four populations in Oman.

Each of 231 (75%) *T*.*annulata* isolates, with a complete set of data, was found to carry multiple genotypes, as several alleles were evident at one or more loci. The mean number of alleles for the ten loci was calculated for each isolate to provide an index value that represented the multiplicity of infection. A summary of multiplicity of infection with respect to the area of isolation is presented in Table 4 and Fig 4. Batinah, Dhofar and Sharqia isolates possessed on average 3.3 to 3.4 alleles per locus, whereas Dhira isolates possessed 2.9. High standard deviation values for each site indicated a significant amount of variance in the data from all sites, with more variation observed in the Dhofar population with a maximum value of 5.6, followed by Batinah with minimum and maximum values of 1.3 and 5.1 (Fig 4).

**Table 4:** Multiplicity of *T*. *annulata* infection in four governorates in Oman.

		Number of allele per locus per isolate				
		n	Mean	Minimum	Maximum	SD
**Population**	**Batinah**	**21**	**3.3**	**1.3**	**5.1**	**1**
	**Dhira**	**57**	**2.9**	**1.6**	**4.7**	**0.8**
	**Dhofar**	**134**	**3.4**	**1.6**	**5.6**	**0.8**
	**Sharqia**	**19**	**3.3**	**2.1**	**4.5**	**0.7**

Of the 231 isolates examined, 131 (58%) had complete data relating to host phenotype (age, sex and breed). *T*. *annulata* isolates in Oman were collected from cattle between 4 and 180 months of age, around 86% of which were female. The majority of cattle were indigenous breeds (71%). Analysis of co-variance (ANCOVA) indicated that there is no apparent association of host variables with multiplicity of infection.

## Discussion

The work reported in this paper is the first detailed genetic analysis of the *T*. *annulata* in Oman. We used a panel of polymorphic micro- and mini-satellite loci, among *T*. *annulata* in four sites in Oman, allowing the inference of genetic parameters driven by neutral processes to define the population genetic structure. The analysis revealed high diversity at each genomic locus, evidence of LD between loci, a high prevalence of multiple genotypes within infected animals and a low level of differentiation between parasites in the four sampling sites.

There were extremely high estimates of genetic diversity within populations from the four sites, both in terms of allelic diversity at each locus studied and in the numbers of co-existing genetically distinct organisms within infected animals. There was a high allelic diversity for the ten loci in all sites, the overall *H*_*e*_ index for each site ranged from 0.816 to 0.854. A similar level of diversity was seen among *T*. *annulata* in other endemic countries [4]. However, a much lower diversity has been reported for *T*. *parva* (*H*_*e*_ ranging between 0.503 and 0.675) in Zambia [28], although a different set of marker was utilised. High diversity can be attributed to a large effective population size (N_e_): i.e. the proportion of the population that contributes to the evolution of genetic diversity [29]. Theoretically, there is a direct relationship between the expected level of diversity and N_e_, so the above observation implies that the effective population size of *T*. *annulata* in Oman is large. However, other factors can contribute to diversity, such as migration and gene flow [30].

The mean numbers of genotypes detected per infected animal ranged between 2.9 and 3.4 genotypes. The number of parasite genotypes found within individual hosts may reflect the transmission intensity and abundance of vector (ticks). Thus, the relatively low multiplicity of infection in Dhira compared to the other three sites, may be linked to variation in tick abundance/species and incidence of parasite infection in ticks, however it is possible that confounding factors such as differences in regional sampling or unknown host-related factors may also explain this finding. Unfortunately, there is no information on abundance and seasonality of ticks in different regions in Oman. Only 2.1% of the variability in multiplicity of infection can be explained by bovine host variables recorded (age, sex and breed). In addition, samples from all sites were collected from apparently healthy animals. The high multiplicity of genotypes among the asymptomatic animals examined in the present study is in accordance with other surveys of clinical cases of *T*. *annulata* and *T*.*parva* in other countries [4, 15]. This is a common feature across many Apicomplexan parasites species infecting human or animal host, which show a high prevalence of mixed genotype infections [31] where the mean number of alleles is related to the intensity of infection in the geographical area.

Mixed genotypes in the bovine host can be considered a pre-requisite for cross-mating and recombination of *T*. *annulata* in the vector. Thus, co-uptake of piroplasm/ gametocytes representing distinct genotypes by the feeding tick may result in cross- fertilisation and the formation of heterozygotes following syngamy. Genetic recombination then occurs in the tick gut leading to the generation of novel genotypes [32, 33]. Assuming random pairing of male and female gametes, the frequency of recombinant types equals the probability that these gametes are sampled from different clones carried in a single animal. Thus, the probability of inbreeding can be related to the numbers of genotypes detected per infection, assuming that all blood form parasites are represented in the gametocyte population [34]. Such an approach has been validated in the malaria parasite [34, 35]. Extrapolating this method for multiplicity of *T*. *annulata* in Oman provides estimates for effective number of clones (ne) of 3.35 and inbreeding of 0.3 (f = 1/ne) [34, 35]. This provides a measure of a high extent of outcrossing of over 50%, which may have important implications for the success of control measures. In particular, new antigenic types, distinct form vaccine genotypes, may be formed and recombination of drug resistant loci may increase the spread of resistance and the risk that multiple drug resistant genotypes will be produced.

Despite the observed extensive genetic diversity, a high level of LD was detected in three of the four *T*. *annulata* populations in Oman. LD can be influenced by diverse factors, other than the extent of inbreeding; including the recombination rate, the local parasite effective population size and population differentiation, caused by subpopulation structure [35]. However, the amount of linkage disequilibrium observed in the present study is much weaker than that expected in the case of a clonal population structure.

Thus, some level of inbreeding is operating in three of our study sites, and mating between different parasite genotypes may not be completely random. Similar to other vector-borne parasites, some degree of spatial structuring in *Theileria* parasites can occur among infected animals, and it may be hypothesised that the extent of clustering depends on epidemiological factors such as the density of ticks, parasite infectivity and genotype-specific immune responses of the host. Currently, no information is available on the extent of transmission in the four sites. However, vertebrate hosts in close proximity can often carry related parasite genotypes that result from crossing, recombination and asexual amplification in a single infected vector [36, 37]. In the case of *Theileria*, this will be true for ticks that fed on the same infected animal. It has been shown that a small number of genetically related parasites can generate significant linkage disequilibrium [38]. Thus, the mild but statistically significant LD exhibited in three of the four *T*. *annulata* populations in Oman may not be related to a clonal pattern of population structure. This is evident by the high diversity of MLGs among isolates in each region implying, as indicated above, a high degree of cross-mating and sexual recombination. Indeed the MLG diversity and estimate of the index of association (Table 2), do not agree with inbreeding being a significant feature of the *T*. *annulata* population structure, as described in bacteria [39]. The high level of heterozygosity, high MOI and high diversity observed in each population would limit the effect of random genetic drift and, therefore, this is also unlikely to explain the observed LD. Such an observation of high genetic diversity and significant genetic linkage disequilibria is consistent with analysis of other *Theileria* populations [4, 28]. Similar patterns of genetic diversity have been observed in other vector-borne parasites such as Plasmodium, and have been attributed to ecological rather than parasite genetic factors [38, 40].

Very low levels of genetic differentiation were detected between *T*. *annulata* parasite populations in the four regions of Oman, with most pair-wise *F*_ST_ values being less than 0.03. In contrast, a moderate to high level of genetic differentiation has been seen among *T*. *parva* parasites between two regions in Zambia, with *F*_ST_ values exceeding 0.10, separated by a similar distance to the sites represented by the Omani populations [28]. This could be related to differences in tick species or intrinsic properties of the two parasites, e.g. rate of gametocyte production and sporozoite infectivity. However, a comparison of the parasite populations of Oman to those in other countries revealed a high level of differentiation between *T*. *annulata* in Oman and Tunisia (F_ST_ = 0.063), and Oman and Turkey (F_ST_ = 0.102_)_. Parasite genotypes in Oman appear to be more closely related to those in Tunisia than Turkey. This suggests that despite ecological and epidemiological barriers between the parasite populations, there is likely to have been slightly more mixing between these populations than between Oman and Turkey; this may be related to the frequency of movement of animals [4] since, in the absence of hosts, ticks migrate over a very restricted range.

Free movement of cattle over a large distance is not a feature in Dhira and Sharqia in Oman. Here animals are kept exclusively on farms, although movement of cattle from one farm to another is not an uncommon practice. It would be of interest to examine parasites in neighboring countries in the region, where theileriosis is also a major problem, to determine how closely related these poplations are, and whether control measures are implemented seperately or if a regional policy can and should be adopted.

**In summary**, analysis of ten putative neutral markers among four populations of *T*. *annulata* in geographically separated regions in Oman revealed a high level of genetic diversity with limited differentiation between regional populations. Broadly, the parasite population appears to be panmictic and, similar to previous studies in *Theileria*, a degree of LD was observed in some of the examined populations. The mild LD observed may be caused by a number of factors, including the over-sampling of related genotypes. A high proportion of the examined animals carried distinct multi-locus genotypes, suggesting a high rate of outcrossing in the tick vector. However, little is known of the interactions among different genotypes and the factors that modulate their transmissibility to the tick. Further investigation of the temporal dynamics of parasite genotypes within infected herds will likely provide a better understanding of complexities of the population structure of *T*. *annulata* as a whole.

## Supporting Information

S1 Table Alleles of 10 micro- and mini-satellite loci identified in 310 *T*. *annulata* isolates from four regional governorates of Oman: Batinah, Dhira, Sharqia, and Dhofar. (DOCX)
