# Supplementary material for: Genetic Diversity and Population Structure of Theileria annulata in Oman
Source: PLoS One. 2015 Oct 15;10(10):e0139581. doi: 10.1371/journal.pone.0139581 (PMC4607491; doi:10.1371/journal.pone.0139581)
Supplement: S1 Table — (DOCX) [file pone.0139581.s001.docx]

**S1: Alleles of 10 micro- and mini-satellite loci among 310 *T. annulata* isolates in four governorates in Oman,** Batinah, Dhira, Sharqia, and Dhofar.

| **populations** | **Ts5** | **Ts6** | **Ts8** | **Ts9** | **Ts12** | **Ts15** | **Ts16** | **Ts20** | **Ts25** | **Ts31** |
| --- | --- | --- | --- | --- | --- | --- | --- | --- | --- | --- |
| Batinah | 262 | 387 | 251 | 366 | 265 | 214 | 353 | 221 | 210 | 257 |
| Batinah | 266 | 369 | 251 | 362 | 261 | 214 | 349 | 239 | 210 | 255 |
| Batinah | 276 | 329 | 277 | 362 | 267 | 214 | 349 | 223 | 218 | 285 |
| Batinah | 262 | 391 | 233 | 362 | 287 | 214 | 349 | 223 | 220 | 267 |
| Batinah | 266 | 377 | 267 | 358 | 329 | 188 | 349 | 223 | 218 | 257 |
| Batinah | 266 | 395 | 263 | 370 | 259 | 240 | 359 | 223 | 218 | 257 |
| Batinah | 266 | 337 | 229 | 358 | 253 | 188 | 369 | 211 | 210 | 333 |
| Batinah | 290 | 429 | 289 | 362 | 293 | 238 | 349 | 221 | 232 | 289 |
| Batinah | 268 | 395 | 283 | 344 | 259 | 214 | 353 | 223 | 220 | 269 |
| Batinah | 268 | 343 | 249 | 354 | 277 | 240 | 355 | 223 | 210 | 235 |
| Batinah | 268 | 403 | 307 | 368 | 285 | 310 | 353 | 221 | 226 | 287 |
| Batinah | 250 | 335 | 297 | 364 | 259 | 240 | 351 | 239 | 214 | 257 |
| Batinah | 256 | 403 | 301 | 378 | 259 | 188 | 355 | 219 | 220 | 287 |
| Batinah | 268 | 253 | 285 | 370 | 259 | 214 | 349 | 227 | 234 | 259 |
| Batinah | 262 | 313 | 213 | 382 | 251 | 188 | 349 | 223 | 218 | 287 |
| Batinah | 266 | 389 | 251 | 364 | 259 | 190 | 353 | 223 | 218 | 295 |
| Batinah | 282 | 363 | 307 | 362 | 259 | 188 | 353 | 223 | 210 | 235 |
| Batinah | 268 | 335 | 257 | 358 | 259 | 262 | 349 | 239 | 220 | 295 |
| Batinah | 268 | 219 | 261 | 362 | 277 | 166 | 457 | 239 | 218 | 285 |
| Batinah | 262 | 393 | 291 | 360 | 259 | 240 | 365 | 223 | 218 | 285 |
| Batinah | 268 | 395 | 233 | 360 | 265 | 190 | 349 | 223 | 218 | 217 |
| Dhira | 266 | 357 | 275 | 378 | 281 | 214 | 355 | 223 | 218 | 287 |
| Dhira | 266 | 307 | 233 | 352 | 259 | 238 | 353 | 223 | 220 | 245 |
| Dhira | 266 | 307 | 251 | 370 | 265 | 214 | 349 | 223 | 242 | 333 |
| Dhira | 266 | 357 | 241 | 352 | 259 | 238 | 353 | 223 | 210 | 295 |
| Dhira | 260 | 307 | 303 | 358 | 259 | 212 | 353 | 223 | 210 | 267 |
| Dhira | 266 | 313 | 211 | 362 | 279 | 238 | 370 | 217 | 218 | 269 |
| Dhira | 266 | 315 | 285 | 364 | 301 | 240 | 349 | 263 | 220 | 307 |
| Dhira | 266 | 371 | 251 | 372 | 259 | 238 | 349 | 223 | 218 | 255 |
| Dhira | 266 | 313 | 299 | 362 | 273 | 238 | 349 | 221 | 210 | 275 |
| Dhira | 266 | 313 | 251 | 378 | 271 | 238 | 359 | 223 | 218 | 267 |
| Dhira | 266 | 313 | 279 | 374 | 271 | 238 | 457 | 223 | 218 | 291 |
| Dhira | 266 | 313 | 211 | 366 | 271 | 238 | 349 | 243 | 218 | 267 |
| Dhira | 260 | 313 | 213 | 362 | 175 | 188 | 349 | 223 | 218 | 257 |
| Dhira | 266 | 377 | 235 | 372 | 259 | 238 | 349 | 223 | 208 | 267 |
| Dhira | 260 | 313 | 237 | 350 | 265 | 188 | 349 | 223 | 210 | 283 |
| Dhira | 266 | 327 | 225 | 348 | 271 | 188 | 349 | 191 | 212 | 279 |
| Dhira | 272 | 319 | 231 | 348 | 267 | 214 | 349 | 223 | 208 | 255 |
| Dhira | 284 | 317 | 309 | 366 | 271 | 330 | 347 | 227 | 214 | 267 |
| Dhira | 262 | 395 | 297 | 364 | 265 | 214 | 347 | 237 | 226 | 265 |
| Dhira | 260 | 313 | 297 | 364 | 259 | 214 | 349 | 191 | 210 | 267 |
| Dhira | 260 | 385 | 231 | 354 | 269 | 238 | 349 | 227 | 212 | 267 |
| Dhira | 284 | 405 | 297 | 362 | 249 | 214 | 349 | 223 | 214 | 287 |
| Dhira | 266 | 369 | 267 | 358 | 249 | 238 | 349 | 223 | 214 | 285 |
| Dhira | 266 | 373 | 265 | 362 | 271 | 238 | 349 | 223 | 214 | 255 |
| Dhira | 266 | 391 | 225 | 362 | 277 | 238 | 457 | 223 | 214 | 313 |
| Dhira | 260 | 375 | 277 | 360 | 271 | 190 | 349 | 223 | 220 | 235 |
| Dhira | 266 | 369 | 301 | 352 | 311 | 238 | 345 | 223 | 218 | 255 |
| Dhira | 266 | 393 | 301 | 364 | 253 | 238 | 349 | 223 | 218 | 293 |
| Dhira | 262 | 391 | 239 | 380 | 277 | 240 | 361 | 223 | 218 | 287 |
| Dhira | 266 | 405 | 301 | 376 | 259 | 188 | 349 | 223 | 210 | 295 |
| Dhira | 262 | 423 | 275 | 364 | 235 | 214 | 341 | 223 | 220 | 275 |
| Dhira | 272 | 357 | 229 | 372 | 265 | 164 | 353 | 223 | 218 | 279 |
| Dhira | 260 | 307 | 297 | 374 | 365 | 238 | 353 | 223 | 210 | 217 |
| Dhira | 278 | 395 | 277 | 374 | 259 | 188 | 341 | 223 | 208 | 249 |
| Dhira | 282 | 307 | 277 | 364 | 303 | 188 | 353 | 227 | 244 | 285 |
| Dhira | 266 | 307 | 263 | 374 | 265 | 212 | 349 | 227 | 218 | 255 |
| Dhira | 266 | 353 | 255 | 374 | 271 | 238 | 351 | 223 | 210 | 341 |
| Dhira | 260 | 393 | 253 | 374 | 271 | 262 | 457 | 227 | 214 | 255 |
| Dhira | 266 | 391 | 283 | 358 | 295 | 188 | 349 | 221 | 208 | 293 |
| Dhira | 264 | 313 | 297 | 360 | 259 | 214 | 355 | 223 | 218 | 331 |
| Dhira | 266 | 313 | 213 | 362 | 253 | 238 | 349 | 223 | 210 | 285 |
| Dhira | 272 | 313 | 263 | 358 | 255 | 188 | 349 | 223 | 214 | 293 |
| Dhira | 260 | 389 | 289 | 366 | 263 | 240 | 349 | 223 | 232 | 237 |
| Dhira | 272 | 383 | 277 | 362 | 271 | 240 | 349 | 223 | 228 | 295 |
| Dhira | 266 | 395 | 283 | 382 | 261 | 240 | 353 | 223 | 224 | 249 |
| Dhira | 266 | 395 | 233 | 352 | 259 | 238 | 349 | 223 | 220 | 217 |
| Dhira | 262 | 385 | 239 | 358 | 255 | 190 | 349 | 227 | 210 | 255 |
| Dhira | 266 | 379 | 279 | 376 | 293 | 240 | 349 | 211 | 214 | 267 |
| Dhira | 262 | 343 | 269 | 350 | 259 | 188 | 347 | 225 | 220 | 275 |
| Dhira | 266 | 313 | 211 | 366 | 295 | 238 | 349 | 223 | 218 | 267 |
| Dhira | 266 | 303 | 277 | 376 | 265 | 262 | 349 | 229 | 208 | 257 |
| Dhira | 260 | 427 | 251 | 370 | 291 | 238 | 349 | 211 | 218 | 267 |
| Dhira | 262 | 369 | 275 | 364 | 261 | 214 | 349 | 223 | 218 | 255 |
| Dhira | 266 | 229 | 211 | 368 | 271 | 238 | 349 | 223 | 218 | 269 |
| Dhira | 272 | 357 | 309 | 372 | 289 | 238 | 349 | 227 | 214 | 293 |
| Dhira | 260 | 395 | 291 | 364 | 259 | 240 | 341 | 223 | 210 | 287 |
| Dhira | 272 | 305 | 295 | 364 | 261 | 240 | 355 | 255 | 210 | 287 |
| Dhofar | 284 | 315 | 213 | 368 | 285 | 188 | 353 | 239 | 218 | 249 |
| Dhofar | 256 | 371 | 297 | 364 | 301 | 190 | 349 | 223 | 210 | 287 |
| Dhofar | 256 | 383 | 241 | 366 | 269 | 264 | 457 | 223 | 210 | 293 |
| Dhofar | 262 | 293 | 263 | 372 | 251 | 240 | 353 | 211 | 210 | 257 |
| Dhofar | 262 | 391 | 263 | 382 | 299 | 214 | 363 | 215 | 238 | 257 |
| Dhofar | 272 | 383 | 213 | 382 | 301 | 190 | 457 | 233 | 228 | 207 |
| Dhofar | 260 | 309 | 213 | 350 | 303 | 286 | 333 | 191 | 218 | 269 |
| Dhofar | 262 | 313 | 275 | 368 | 245 | 214 | 349 | 223 | 210 | 217 |
| Dhofar | 278 | 383 | 289 | 374 | 255 | 262 | 349 | 223 | 218 | 291 |
| Dhofar | 268 | 313 | 213 | 366 | 291 | 240 | 355 | 211 | 218 | 307 |
| Dhofar | 268 | 315 | 213 | 368 | 257 | 188 | 349 | 223 | 210 | 287 |
| Dhofar | 268 | 405 | 213 | 348 | 273 | 188 | 351 | 231 | 210 | 267 |
| Dhofar | 266 | 313 | 213 | 362 | 257 | 190 | 349 | 223 | 218 | 217 |
| Dhofar | 260 | 353 | 281 | 362 | 349 | 190 | 365 | 221 | 218 | 327 |
| Dhofar | 266 | 313 | 275 | 368 | 265 | 214 | 349 | 225 | 210 | 295 |
| Dhofar | 266 | 315 | 213 | 364 | 245 | 240 | 349 | 225 | 218 | 293 |
| Dhofar | 266 | 313 | 213 | 362 | 267 | 240 | 349 | 223 | 218 | 293 |
| Dhofar | 292 | 315 | 275 | 368 | 265 | 190 | 353 | 227 | 218 | 217 |
| Dhofar | 268 | 313 | 213 | 360 | 253 | 188 | 349 | 223 | 220 | 217 |
| Dhofar | 268 | 315 | 275 | 362 | 273 | 262 | 349 | 227 | 218 | 217 |
| Dhofar | 284 | 383 | 263 | 366 | 279 | 214 | 349 | 223 | 218 | 257 |
| Dhofar | 266 | 315 | 263 | 362 | 279 | 262 | 349 | 221 | 210 | 287 |
| Dhofar | 266 | 313 | 263 | 362 | 279 | 188 | 353 | 227 | 218 | 247 |
| Dhofar | 262 | 357 | 263 | 360 | 273 | 240 | 355 | 225 | 220 | 217 |
| Dhofar | 266 | 315 | 215 | 348 | 295 | 262 | 353 | 223 | 210 | 251 |
| Dhofar | 266 | 371 | 251 | 362 | 257 | 188 | 349 | 223 | 220 | 217 |
| Dhofar | 266 | 313 | 219 | 374 | 279 | 214 | 349 | 223 | 228 | 207 |
| Dhofar | 256 | 315 | 213 | 362 | 295 | 188 | 349 | 223 | 218 | 259 |
| Dhofar | 266 | 315 | 213 | 358 | 281 | 188 | 355 | 223 | 210 | 217 |
| Dhofar | 266 | 315 | 269 | 362 | 287 | 188 | 349 | 223 | 210 | 293 |
| Dhofar | 266 | 315 | 213 | 366 | 249 | 264 | 457 | 223 | 220 | 207 |
| Dhofar | 266 | 315 | 263 | 362 | 295 | 188 | 353 | 223 | 210 | 269 |
| Dhofar | 266 | 315 | 255 | 376 | 343 | 188 | 349 | 223 | 214 | 207 |
| Dhofar | 262 | 315 | 275 | 370 | 175 | 240 | 353 | 223 | 210 | 217 |
| Dhofar | 266 | 313 | 287 | 346 | 311 | 190 | 349 | 223 | 220 | 275 |
| Dhofar | 260 | 315 | 239 | 360 | 323 | 240 | 343 | 227 | 218 | 267 |
| Dhofar | 272 | 313 | 263 | 372 | 267 | 188 | 457 | 227 | 210 | 217 |
| Dhofar | 262 | 315 | 285 | 366 | 273 | 262 | 353 | 227 | 210 | 267 |
| Dhofar | 262 | 315 | 287 | 372 | 325 | 214 | 359 | 227 | 220 | 249 |
| Dhofar | 268 | 315 | 287 | 346 | 265 | 214 | 349 | 227 | 214 | 275 |
| Dhofar | 262 | 309 | 281 | 350 | 261 | 286 | 359 | 315 | 252 | 269 |
| Dhofar | 272 | 315 | 275 | 362 | 257 | 214 | 333 | 239 | 220 | 267 |
| Dhofar | 262 | 403 | 213 | 392 | 301 | 214 | 349 | 227 | 220 | 217 |
| Dhofar | 266 | 313 | 275 | 362 | 251 | 214 | 349 | 227 | 216 | 307 |
| Dhofar | 262 | 315 | 263 | 362 | 253 | 262 | 343 | 223 | 218 | 293 |
| Dhofar | 262 | 315 | 263 | 350 | 295 | 262 | 353 | 223 | 218 | 217 |
| Dhofar | 266 | 315 | 263 | 364 | 301 | 214 | 349 | 223 | 218 | 293 |
| Dhofar | 268 | 315 | 213 | 364 | 257 | 190 | 457 | 223 | 218 | 247 |
| Dhofar | 266 | 315 | 251 | 364 | 257 | 190 | 457 | 223 | 218 | 287 |
| Dhofar | 266 | 315 | 263 | 366 | 257 | 240 | 349 | 227 | 218 | 293 |
| Dhofar | 266 | 315 | 263 | 366 | 253 | 240 | 349 | 223 | 218 | 257 |
| Dhofar | 268 | 313 | 213 | 360 | 285 | 188 | 333 | 245 | 210 | 217 |
| Dhofar | 266 | 315 | 213 | 362 | 279 | 188 | 349 | 223 | 210 | 217 |
| Dhofar | 266 | 315 | 263 | 378 | 295 | 188 | 353 | 227 | 218 | 293 |
| Dhofar | 266 | 313 | 213 | 372 | 257 | 164 | 353 | 223 | 210 | 267 |
| Dhofar | 280 | 313 | 263 | 366 | 257 | 240 | 353 | 225 | 210 | 293 |
| Dhofar | 256 | 309 | 275 | 372 | 247 | 240 | 349 | 235 | 210 | 217 |
| Dhofar | 272 | 315 | 289 | 366 | 305 | 240 | 349 | 227 | 220 | 217 |
| Dhofar | 262 | 313 | 275 | 364 | 257 | 190 | 353 | 227 | 210 | 293 |
| Dhofar | 268 | 355 | 225 | 388 | 347 | 214 | 349 | 191 | 210 | 327 |
| Dhofar | 268 | 315 | 263 | 374 | 301 | 188 | 349 | 223 | 218 | 217 |
| Dhofar | 262 | 313 | 263 | 366 | 257 | 164 | 353 | 225 | 210 | 293 |
| Dhofar | 284 | 313 | 263 | 366 | 249 | 240 | 353 | 223 | 218 | 217 |
| Dhofar | 278 | 315 | 263 | 366 | 257 | 214 | 355 | 227 | 210 | 217 |
| Dhofar | 272 | 315 | 213 | 362 | 279 | 240 | 349 | 211 | 210 | 217 |
| Dhofar | 262 | 403 | 213 | 382 | 257 | 286 | 349 | 223 | 210 | 285 |
| Dhofar | 268 | 421 | 263 | 362 | 267 | 214 | 349 | 223 | 210 | 247 |
| Dhofar | 272 | 315 | 285 | 366 | 301 | 188 | 457 | 221 | 218 | 235 |
| Dhofar | 268 | 383 | 263 | 366 | 273 | 214 | 457 | 223 | 210 | 217 |
| Dhofar | 266 | 383 | 263 | 366 | 279 | 214 | 457 | 223 | 210 | 217 |
| Dhofar | 266 | 315 | 275 | 364 | 259 | 264 | 349 | 223 | 228 | 215 |
| Dhofar | 266 | 313 | 225 | 366 | 301 | 240 | 349 | 221 | 220 | 287 |
| Dhofar | 268 | 313 | 215 | 378 | 257 | 264 | 349 | 251 | 232 | 275 |
| Dhofar | 268 | 405 | 281 | 354 | 267 | 214 | 363 | 241 | 220 | 293 |
| Dhofar | 262 | 315 | 225 | 364 | 301 | 240 | 349 | 223 | 218 | 293 |
| Dhofar | 262 | 315 | 309 | 366 | 267 | 214 | 349 | 223 | 218 | 275 |
| Dhofar | 266 | 315 | 285 | 362 | 253 | 214 | 349 | 223 | 216 | 257 |
| Dhofar | 266 | 313 | 263 | 366 | 271 | 240 | 351 | 223 | 218 | 217 |
| Dhofar | 256 | 417 | 297 | 364 | 283 | 356 | 361 | 227 | 256 | 249 |
| Dhofar | 266 | 393 | 289 | 364 | 245 | 238 | 353 | 223 | 210 | 235 |
| Dhofar | 262 | 369 | 287 | 372 | 259 | 240 | 349 | 211 | 218 | 269 |
| Dhofar | 272 | 383 | 297 | 368 | 259 | 240 | 349 | 223 | 218 | 257 |
| Dhofar | 268 | 395 | 297 | 364 | 259 | 214 | 349 | 223 | 218 | 257 |
| Dhofar | 266 | 405 | 213 | 362 | 263 | 188 | 349 | 223 | 218 | 293 |
| Dhofar | 256 | 415 | 267 | 376 | 283 | 356 | 361 | 225 | 210 | 251 |
| Dhofar | 266 | 353 | 239 | 364 | 295 | 214 | 353 | 225 | 214 | 251 |
| Dhofar | 284 | 357 | 213 | 376 | 295 | 264 | 361 | 227 | 210 | 217 |
| Dhofar | 272 | 389 | 271 | 364 | 291 | 214 | 353 | 227 | 218 | 259 |
| Dhofar | 262 | 389 | 271 | 364 | 289 | 214 | 353 | 223 | 218 | 269 |
| Dhofar | 284 | 315 | 263 | 388 | 295 | 262 | 349 | 223 | 220 | 247 |
| Dhofar | 256 | 315 | 213 | 348 | 301 | 214 | 349 | 223 | 218 | 247 |
| Dhofar | 284 | 371 | 263 | 364 | 279 | 188 | 349 | 235 | 218 | 249 |
| Dhofar | 268 | 315 | 213 | 378 | 273 | 214 | 349 | 223 | 210 | 293 |
| Dhofar | 256 | 357 | 263 | 366 | 327 | 238 | 349 | 223 | 218 | 293 |
| Dhofar | 266 | 315 | 275 | 362 | 253 | 214 | 353 | 227 | 210 | 293 |
| Dhofar | 284 | 309 | 213 | 350 | 251 | 240 | 363 | 211 | 210 | 217 |
| Dhofar | 266 | 313 | 225 | 368 | 295 | 240 | 353 | 223 | 210 | 293 |
| Dhofar | 266 | 405 | 213 | 370 | 287 | 214 | 349 | 223 | 234 | 367 |
| Dhofar | 262 | 315 | 239 | 352 | 253 | 188 | 353 | 227 | 210 | 293 |
| Dhofar | 262 | 337 | 289 | 368 | 249 | 212 | 359 | 223 | 214 | 217 |
| Dhofar | 268 | 313 | 255 | 360 | 279 | 164 | 349 | 221 | 218 | 217 |
| Dhofar | 262 | 371 | 289 | 368 | 257 | 264 | 349 | 229 | 252 | 251 |
| Dhofar | 266 | 313 | 251 | 366 | 257 | 164 | 349 | 219 | 218 | 293 |
| Dhofar | 266 | 313 | 263 | 360 | 279 | 164 | 349 | 223 | 210 | 267 |
| Dhofar | 262 | 339 | 275 | 360 | 257 | 190 | 347 | 223 | 210 | 295 |
| Dhofar | 268 | 315 | 213 | 356 | 257 | 164 | 355 | 221 | 210 | 217 |
| Dhofar | 290 | 315 | 277 | 370 | 257 | 264 | 349 | 223 | 218 | 251 |
| Dhofar | 262 | 337 | 243 | 362 | 279 | 188 | 349 | 227 | 214 | 251 |
| Dhofar | 266 | 315 | 263 | 376 | 279 | 262 | 349 | 223 | 220 | 217 |
| Dhofar | 266 | 337 | 241 | 372 | 279 | 262 | 347 | 227 | 216 | 205 |
| Dhofar | 266 | 313 | 297 | 374 | 253 | 214 | 355 | 227 | 210 | 217 |
| Dhofar | 266 | 313 | 263 | 350 | 301 | 264 | 351 | 239 | 218 | 267 |
| Dhofar | 266 | 383 | 213 | 380 | 301 | 264 | 349 | 223 | 210 | 293 |
| Dhofar | 262 | 315 | 263 | 368 | 295 | 188 | 349 | 227 | 218 | 217 |
| Dhofar | 266 | 315 | 263 | 366 | 279 | 214 | 353 | 223 | 210 | 217 |
| Dhofar | 262 | 313 | 213 | 298 | 253 | 190 | 353 | 227 | 218 | 293 |
| Dhofar | 266 | 315 | 265 | 364 | 271 | 166 | 353 | 221 | 210 | 303 |
| Dhofar | 266 | 315 | 275 | 384 | 253 | 264 | 353 | 227 | 218 | 293 |
| Dhofar | 268 | 353 | 257 | 366 | 273 | 214 | 349 | 199 | 228 | 257 |
| Dhofar | 266 | 315 | 219 | 362 | 315 | 214 | 353 | 223 | 218 | 293 |
| Dhofar | 266 | 309 | 263 | 366 | 279 | 214 | 457 | 223 | 218 | 293 |
| Dhofar | 262 | 315 | 229 | 366 | 301 | 240 | 365 | 223 | 220 | 293 |
| Dhofar | 262 | 313 | 275 | 380 | 271 | 188 | 353 | 235 | 232 | 217 |
| Dhofar | 284 | 369 | 251 | 366 | 295 | 262 | 349 | 351 | 220 | 257 |
| Dhofar | 262 | 315 | 277 | 366 | 299 | 188 | 347 | 223 | 210 | 293 |
| Dhofar | 262 | 383 | 277 | 366 | 279 | 190 | 349 | 227 | 220 | 217 |
| Dhofar | 266 | 395 | 285 | 372 | 257 | 262 | 353 | 223 | 218 | 267 |
| Dhofar | 268 | 315 | 249 | 352 | 309 | 240 | 349 | 215 | 210 | 267 |
| Dhofar | 272 | 461 | 263 | 386 | 291 | 214 | 359 | 223 | 226 | 257 |
| Dhofar | 266 | 307 | 253 | 366 | 279 | 264 | 365 | 223 | 210 | 293 |
| Dhofar | 266 | 337 | 287 | 364 | 279 | 214 | 347 | 221 | 218 | 287 |
| Dhofar | 272 | 307 | 249 | 364 | 279 | 190 | 349 | 191 | 214 | 287 |
| Dhofar | 268 | 315 | 263 | 382 | 279 | 240 | 457 | 227 | 210 | 293 |
| Dhofar | 266 | 365 | 275 | 368 | 295 | 190 | 349 | 227 | 210 | 293 |
| Sharqia | 272 | 315 | 185 | 370 | 175 | 262 | 345 | 223 | 230 | 257 |
| Sharqia | 266 | 313 | 299 | 372 | 259 | 188 | 347 | 223 | 218 | 257 |
| Sharqia | 266 | 315 | 297 | 370 | 259 | 188 | 349 | 223 | 210 | 267 |
| Sharqia | 266 | 307 | 269 | 358 | 295 | 240 | 347 | 227 | 214 | 257 |
| Sharqia | 266 | 313 | 297 | 348 | 259 | 188 | 347 | 223 | 220 | 257 |
| Sharqia | 284 | 367 | 277 | 362 | 251 | 238 | 345 | 223 | 218 | 283 |
| Sharqia | 266 | 405 | 243 | 358 | 265 | 164 | 349 | 223 | 218 | 249 |
| Sharqia | 262 | 315 | 233 | 366 | 259 | 164 | 355 | 223 | 212 | 255 |
| Sharqia | 266 | 379 | 283 | 378 | 311 | 240 | 383 | 223 | 210 | 287 |
| Sharqia | 266 | 383 | 265 | 362 | 249 | 188 | 355 | 223 | 220 | 257 |
| Sharqia | 274 | 323 | 267 | 368 | 267 | 190 | 383 | 223 | 210 | 257 |
| Sharqia | 272 | 355 | 313 | 360 | 325 | 166 | 353 | 227 | 220 | 293 |
| Sharqia | 260 | 313 | 313 | 372 | 299 | 212 | 353 | 223 | 218 | 257 |
| Sharqia | 262 | 395 | 265 | 364 | 311 | 188 | 457 | 223 | 210 | 287 |
| Sharqia | 272 | 315 | 265 | 364 | 259 | 188 | 349 | 239 | 210 | 293 |
| Sharqia | 272 | 315 | 277 | 376 | 259 | 214 | 355 | 225 | 214 | 269 |
| Sharqia | 272 | 307 | 275 | 368 | 265 | 212 | 457 | 223 | 214 | 259 |
| Sharqia | 284 | 413 | 247 | 374 | 295 | 212 | 457 | 227 | 218 | 287 |
| Sharqia | 266 | 395 | 235 | 370 | 259 | 188 | 457 | 223 | 220 | 259 |

**mm**
